# Reply: Can safety and innovation coexist in the global expansion of laser balloon ablation?

**DOI:** 10.1016/j.hroo.2025.08.037

**Published:** 2025-09-02

**Authors:** Ken Kawase, Reina Tonegawa-Kuji, Kengo Kusano

**Affiliations:** 1 Department of Cardiovascular Medicine, National Cerebral and Cardiovascular Center, Suita, Japan; 2 Department of Advanced Cardiovascular Medicine, Graduate School of Medical Sciences, Kumamoto University, Kumamoto, Japan; 3 Department of Medical and Health Information Management, National Cerebral and Cardiovascular Center, Suita, Japan

We sincerely appreciate the thoughtful comments on our article.^1^

Our findings, although based on Japanese data, can be interpreted in a global context. Variations in health care infrastructure, institutional resources, and operator experience significantly influence the feasibility and safety of ablation technologies. Even within Japan, national surveys show wide variation in ablation rates across regions, linked to disparities in arrhythmia specialists, access to high-volume centers, and socioeconomic factors.^2^

Similar heterogeneity has been observed in the United States. A nationwide analysis of more than 50,000 procedures also demonstrated regional disparities in complications and hospital stay, influenced by hospital size, teaching status, and patient characteristics.^3^

Cost-effectiveness data from the The Catheter Ablation vs Antiarrhythmic Drug Therapy for Atrial Fibrillation (CABANA) trial further emphasize the relevance of health care context. In countries with lower procedural costs or stricter thresholds for economic value, such as many emerging economies, catheter ablation may be more favorable than in higher-cost systems. These findings stress the need for regionally tailored implementation strategies.^4^

International collaboration will be essential to validate these observations and to establish globally relevant guidance. Given the variety of available ablation technologies, including radiofrequency, cryoballoon, pulsed field, and laser balloon, device selection should be aligned with each country’s clinical environment, infrastructure, and operator expertise.

Taken together, these studies underscore that institutional capacity and health system characteristics significantly affect ablation outcomes. We hope our findings support practical, context-sensitive decisions for the safe and effective use of evolving technologies.

Once again, we thank the author for recognizing the broader relevance of our work.
